# Sleep benefits different stages of memory in *Drosophila*

**DOI:** 10.3389/fphys.2023.1087025

**Published:** 2023-01-19

**Authors:** Katie Marquand, Camilla Roselli, Isaac Cervantes-Sandoval, Tamara Boto

**Affiliations:** ^1^ Department of Physiology, School of Medicine, Trinity College Institute of Neuroscience, Trinity College Dublin, Dublin, Ireland; ^2^ Trinity College Institute of Neuroscience, School of Genetics and Microbiology, Smurfit Institute of Genetics and School of Natural Sciences, Trinity College Dublin, Dublin, Ireland; ^3^ Department of Biology, Georgetown University, Washington, DC, United States; ^4^ Interdisciplinary Program in Neuroscience, Georgetown University, Washington, DC, United States

**Keywords:** Drosophila, memory, sleep, mushroom body, fan shaped body, dopamine

## Abstract

Understanding the physiological mechanisms that modulate memory acquisition and consolidation remains among the most ambitious questions in neuroscience. Massive efforts have been dedicated to deciphering how experience affects behavior, and how different physiological and sensory phenomena modulate memory. Our ability to encode, consolidate and retrieve memories depends on internal drives, and sleep stands out among the physiological processes that affect memory: one of the most relatable benefits of sleep is the aiding of memory that occurs in order to both prepare the brain to learn new information, and after a learning task, to consolidate those new memories. *Drosophila* lends itself to the study of the interactions between memory and sleep. The fruit fly provides incomparable genetic resources, a mapped connectome, and an existing framework of knowledge on the molecular, cellular, and circuit mechanisms of memory and sleep, making the fruit fly a remarkable model to decipher the sophisticated regulation of learning and memory by the quantity and quality of sleep. Research in *Drosophila* has stablished not only that sleep facilitates learning in wild-type and memory-impaired animals, but that sleep deprivation interferes with the acquisition of new memories. In addition, it is well-accepted that sleep is paramount in memory consolidation processes. Finally, studies in *Drosophila* have shown that that learning itself can promote sleep drive. Nevertheless, the molecular and network mechanisms underlying this intertwined relationship are still evasive. Recent remarkable work has shed light on the neural substrates that mediate sleep-dependent memory consolidation. In a similar way, the mechanistic insights of the neural switch control between sleep-dependent and sleep-independent consolidation strategies were recently described. This review will discuss the regulation of memory by sleep in *Drosophila*, focusing on the most recent advances in the field and pointing out questions awaiting to be investigated.

## 1 Introduction

Memory is essential for survival. Animals need to be able to learn from and remember past experiences to inform future decisions and guide behavior. In the laboratory, *Drosophila melanogaster* can learn to approach odors that have been previously paired with a reward, such as food, or to avoid odors that have been paired with an aversive stimulus such as an electric shock (Tully and Quinn, 1985; Busto et al., 2010). The circuits and neural mechanisms involved in creating these behavioral responses are complex. One key region of the *Drosophila* brain involved in olfactory, taste, visual and courtship memory, is the mushroom body (MB) (Heisenberg et al., 1985; McBride et al., 1999; Vogt et al., 2014; Masek and Keene, 2016; Boto et al., 2020). In the case of associative olfactory memory, odorant molecules bind to receptors in the antennae and activate neurons in the antennal lobe, which then send olfactory information to the MB via projection neurons (PN) (Davis, 2005; Fiala, 2007; Su et al., 2009; Martin et al., 2013). These PNs relay olfactory information to a small subset of the ∼2,200 Kenyon cells (KCs) in the MB calyx, creating sparse representations of numerous different odors (Jefferis et al., 2007; Turner et al., 2008; Aso et al., 2014a; Lin et al., 2014). In the case of taste memory, contact with appetitive taste induces the proboscis extension reflex (PER) (Dethier, 1976). When the attractive taste (such as sucrose) is paired with a punishing stimulus such as bitter taste or heat, flies exhibit an avoidance response that inhibits PER. Taste information is also relayed to the MB by neurons projecting from the subesophageal zone (SEZ), and conditioned taste responses depend on KC and MB circuitry (Kirkhart and Scott, 2015; Masek et al., 2015; Masek and Keene, 2016). KCs are classified into three main subtypes: α/β, α’/β’, and γ (Crittenden et al., 1998; Tanaka et al., 2008; Aso et al., 2014a; Guven-Ozkan and Davis, 2014). The axons of these KCs form the MB lobes, innervated by mushroom body output neurons (MBONs) which then project to different areas in the protocerebrum and mediate the behavioral output (Tanaka et al., 2008; Aso et al., 2014a). Dopaminergic neurons (DANs) act as modulators of synaptic weight and innervate discrete compartments of the MB (Tanaka et al., 2008). These compartments receive input from different types of DANs and contain dendrites of corresponding MBON types (Tanaka et al., 2008; Mao and Davis, 2009; Aso et al., 2014a; Aso et al., 2014b). There are two clusters of DANs which innervate the MB lobes: PPL1 which innervates the vertical lobes (α, α’), responds strongly to aversive stimuli and is necessary for aversive conditioning (Riemensperger et al., 2005; Mao and Davis, 2009; Galili et al., 2014; Cervantes-Sandoval et al., 2017), and PAM which innervates the horizontal lobes (β, β’, γ), responds to rewarding stimuli and is necessary for appetitive conditioning (Liu C. et al., 2012; Yamagata et al., 2015; Yamagata et al., 2016). The input from these modulatory DANs alters the synaptic weights between KCs and MBONs to induce the appropriate behavioral response to a presented odor (Séjourné et al., 2011; Aso et al., 2014b; Hige et al., 2015; Owald et al., 2015; Perisse et al., 2016; Felsenberg et al., 2018). A third group of dopaminergic neurons, PPL2, innervates the MB calyx and has been shown to shape olfactory responses in the KCs and modulate memory strength (Mao and Davis, 2009; Boto et al., 2019). In addition to this complex circuitry, MB activity is modulated by other extrinsic neurons, some of which innervate widely the MB neuropile and whose activity is necessary for efficient memory acquisition, consolidation, and retrieval (Yu et al., 2005; Liu and Davis, 2009; Cervantes-Sandoval and Davis, 2012; Lin et al., 2014; Amin et al., 2020; Baltruschat et al., 2021; Prisco et al., 2021).

In the brain of *Drosophila* we can find structures other than the MB mediating different types of memory. The central complex encompasses the ellipsoid body (EB) and the fan shaped body (FB). The EB has been involved in long-term memory consolidation, visual recognition, and spatial orientation memory (Wu et al., 2007; Neuser et al., 2008; Pan et al., 2009; Zhang et al., 2013; Guo et al., 2015). Interestingly, a subset of EB neurons are also involved in generating sleep drive (Liu et al., 2016; Donlea et al., 2018; Shafer and Keene, 2021; Andreani et al., 2022). The FB has been implicated in visual learning tasks and conditioned nociceptive avoidance (Liu et al., 2006; Wang et al., 2008; Pan et al., 2009; Hu et al., 2018). The FB is a layered structure in the central brain with critical behavioral functions like locomotion (Strauss, 2002) and, importantly, sleep. For example, the FB has been shown to play a role in regulating quiescence (Donlea et al., 2011) and sleep promoting neurons projecting to the FB convey the output of sleep homeostasis (Donlea et al., 2014).

However, the aforementioned central complexes are not the only circuits involved in sleep. As such, peripheral neurons are becoming more relevant in the study of sleep regulation (Seidner et al., 2015; Jones et al., 2022) Additionally, it was shown that some drivers traditionally used to implicate central neurons in sleep homeostasis affect peripheral neurons, arguing that sleep could be indeed regulated by those and not only by central complexes (Satterfield et al., 2022). Glia has also been implicated in sleep regulation (Artiushin et al., 2018; Blum et al., 2021). The inhibition of *Appl*, the *Drosophila* homolog of amyloid precursor protein, in astrocyte-like and cortex glia resulted in increased sleep and longer sleep bouts, whereas overexpression of *Appl* lead to the opposite effect (Farca Luna et al., 2017). The sleep phenotype induced by *Appl* inhibition could be rescued by increasing the expression of the glutamate transporter dEaat1 (Farca Luna et al., 2017) and, interestingly, Eaat1 is required for the consolidation of long-term memories (Matsuno et al., 2019). Glia has an established role in neural metabolism, providing neurons with energy during times of high energy demand, such as long-term memory formation (de Tredern et al., 2021; De Backer and Grunwald Kadow, 2022; Silva et al., 2022). Although it is likely that the roles of glia in sleep and memory are intertwined, this relationship is currently largely unexplored.

Sleep is conserved across species, and it is considered indispensable for health. As in other animals, in *Drosophila* there are two main processes involved in regulating sleep: a circadian component which regulates the sleep-wake cycles, and a homeostatic component which reacts to internal changes that alter the “need for sleep”, for example, increasing sleep duration after sleep deprivation (Borbély, 1982). In *Drosophila*, the definition of sleep is based on behavioral criteria: quiescence, increased arousal threshold, change in posture, and inactivity of at least 5 min (Hendricks et al., 2000; Shaw et al., 2000). Previous work has phenomenally reflected on the literature related to sleep in *Drosophila*, its neural substrates, and novel approaches and research in the field (Dubowy and Sehgal, 2017; Dissel, 2020; Dissel et al., 2020; Weiss and Donlea, 2021a; Gilestro, 2021; Shafer and Keene, 2021) while the present work will aim to describe recent findings on the specific modulation of memory by sleep.

Perhaps attesting the complexity of memory systems, sleep is one of the most relevant biological processes that gates and stabilises memory; a relationship that can be easily demonstrated by the relatable detrimental effects that sleep deprivation has in cognitive abilities. Nonetheless, the precise mechanisms by which sleep affects memory remain largely elusive. The two, however, rely on some overlapping circuitry in the brain, which provides some clues as to how this relationship is mechanistically modulated. Considering the importance of sleep in cognitive functions, it is undeniable that the aforementioned FB plays a key role in enabling and improving memories in *Drosophila*. Similarly, the MBs have been implicated in sleep regulation (Joiner et al., 2006; Sitaraman et al., 2015a; Sitaraman et al., 2015b) as, at least in part, the arousal effects of dopamine rely on the innervation of dopaminergic neurons in the MB (Sitaraman et al., 2015b; Driscoll et al., 2020; Driscoll et al., 2021). These dopaminergic neurons can be the same neurons that enable memory and seem to have a dual role in regulating sleep and arousal (Andretic et al., 2005; Kume et al., 2005; Lebestky et al., 2009). Administration of a tyrosine hydroxylase inhibitor increases sleep duration, and, conversely, administering psychostimulants to increase dopamine signalling decreases sleep (Ueno et al., 2012). The D1-like dopamine receptor DA1 (Dop1R1) is essential for memory formation (Kim et al., 2007), whereas it has also been reported to mediate wakefulness through caffeine (Andretic et al., 2008) or startle-induced arousal (Lebestky et al., 2009), and DA1 in the dorsal FB mediates the arousal effect of dopamine (Ueno et al., 2012). Moreover, the specific dopamine neurons innervating the dorsal FB to promote wakefulness include the aforementioned PPL1 cluster (Liu Q. et al., 2012). This data together highlight that the circuits and molecular mechanisms for both memory and sleep are closely intertwined. In this article we will review findings on the relationship between memory and sleep, looking at cognitive effects of sleep deprivation and induced sleep, and detailing what we know about the molecular and circuit level structures that modulate this relationship.

## 2 Memory acquisition is impaired by sleep deprivation

A common method to investigate the role that sleep plays in the formation of memories is to evaluate the performance of short-sleeping animals in memory assays. One such assay is the heat-box paradigm, in which half of a chamber is heated to 39°C and over a training period flies exposed to this environment learn to avoid the heated area (Wustmann et al., 1996). The memory persists in wild-type flies, and they continue to avoid the previously heated area even after it has returned to a preferable temperature. However, loss-of-function mutations in *Hyperkinetic*, a gene encoding a modulatory element of the Shaker channel, translate into decreased sleep, possibly due to changes in neural excitability. Although these flies display learning, they quickly lose their bias towards the unheated side, showing a detriment of short-term memory (Bushey et al., 2007).

There are also numerous lines of evidence of the supportive role of sleep in memory based on sleep deprivation experiments. Sleep deprivation can be achieved through mechanical stimulation using different apparatus: the SNAP, or Sleep Nullifying Apparatus which involves tilting tubes housing flies such that the sleeping flies are displaced (Melnattur et al., 2020), the SLIDE, Sleep Interrupting Device, which utilises a treadmill-like floor to force the flies to continuously walk (Seugnet et al., 2008), a system which rotates the vials along their major axis before dropping it a short distance (Li et al., 2009), or the DART, *Drosophila* ARousal Tracking system, which video tracks the flies behaviour and utilises a motor to inflict a gentle but unpredictable vibrating stimulus which disrupts the sleep pattern (Faville et al., 2015). More recently, a system was developed where flies were monitored, and upon 20 s of immobility, the housing tube would rotate gently, waking the fly up (Geissmann et al., 2017). This system implies a much gentler stimulation, and surprisingly reported milder effects of sleep deprivation in overall health.

Sleep deprivation using the mechanical approaches described above has proven to be detrimental for memory in different memory and learning assays, like aversive olfactory conditioning (Li et al., 2009). During this task flies are exposed sequentially to two odours, one being paired with an aversive stimulus such as an electric shock. To measure memory retention, flies are typically transferred after training to the choice point in a T-maze and forced to choose between the two odours they were previously exposed to (Busto et al., 2010). Memory scores will represent the proportion of flies that avoid the arm with the odour previously paired with the shock (Tully and Quinn, 1985). Depending on the protocol of training trials and the time between training and testing phases, short-term memory (STM) or long-term memory (LTM) can be evaluated (Margulies et al., 2005). One day of sleep deprivation before training leads to deficits in short-term olfactory memory (Li et al., 2009). Although the authors did not find differences in memory acquisition between sleep deprived and control flies, 1-h after training the memory scores were significantly decreased in flies that had been sleep deprived. Sleep deprivation also impairs memory in the Aversive Phototaxic Suppression (APS) assay (Seugnet et al., 2008). During this task, flies are placed in a T-maze and given a choice between a darkened or light tube. Under naïve circumstances, flies exhibit an innate attraction towards the light, but when the light tube is reinforced with an aversive stimulus, such as quinine, they learn to avoid it (Le Bourg and Buecher, 2002). When wild-type flies were forced to stay awake for 6 h before testing, there was a significant decrease in performance on this task (Seugnet et al., 2008). Learning could be restored quickly following only 2 h of recovery sleep, suggesting that one bout of sleep deprivation does not cause long-term detrimental effects (Seugnet et al., 2008). However, during early life in *Drosophila* there is a higher sleep demand. Sleep is necessary for development, highlighted by the fact that sleep deprivation on the first day of adulthood leads to long-term learning deficits in the APS task even after 3 days of recovery (Seugnet et al., 2011). It is suggested that these deficits are caused by reduced dopamine signalling as a consequence of increased transcript levels of DA1 (Seugnet et al., 2011). This day one sleep deprivation also causes deficits in other learning paradigms, including courtship conditioning, even after 3 days of recovery (Seugnet et al., 2011).

More recently, a similar performance detriment following sleep deprivation was observed in a spatial learning assay modelled after the classic Morris Water Maze (Melnattur et al., 2021). Here, wild-type flies were tasked with locating a ‘cold tile’ in a thermal maze by learning the association with a visual cue. Under regular sleep conditions, wild-type flies became increasingly fast at locating the tile, reducing their time by 80% over 10 trials. This indicates that they have learnt the relevant association between the visual cue and the location of the tile. However, following a night of sleep deprivation using the SNAP, wild-type flies became significantly impaired performing in this task, with practically abolished learning (Melnattur et al., 2021). Old age is associated with a decrease in dopamine, a decrease in sleep, and general memory impairments and this age-dependent cognitive decline is also seen in flies (Iliadi and Boulianne, 2010). 21–24 day old flies show a decrease in total sleep compared to 4–5 day old flies and also display impairments in spatial learning (Melnattur et al., 2021). However, age-dependent cognitive decline in spatial learning can be restored by increasing dopaminergic signalling through DA1 receptors, specifically in EB, or restored by enhancing sleep (Melnattur et al., 2021).

Sleep deprivation can also be achieved by thermogenic activation of selected neuronal subsets. Driving sleep deprivation by thermogenic activation of a subset of peripheral *pickpocket* (*ppk*)-expressing neurons and octopaminergic neurons causes impairment of short-term aversive taste memory (Seidner et al., 2015). This impairment can be rescued if the animals, after thermogenic activation of the neurons, are allowed to recover for 3 h prior training and testing, suggesting that the recovery period allows the formation of STM previously disrupted (Seidner et al., 2015).

In a novel appetitive operant conditioning task flies were rewarded with sucrose when they turned in the desired direction on a Y-shaped track (Wiggin et al., 2021). It was reported that only flies who rested during training (with rest defined as 1 min or more of continuous inactivity) were able to learn and perform well in this task. Interestingly, and perhaps counter-intuitively, optogenetically inducing sleep via activation of the dorsal FB did not enhance learning (Wiggin et al., 2021). This suggests that spontaneous rest is required to perform well but not sufficient to induce learning in this task, or that the specific restorative rest that gates learning in this operant task is driven by a different neural population. In this operant task, the individual flies who tended to rest during training and therefore performed well at the task also spent more time adjacent to the sucrose reward, indicating an increased intake of food (Wiggin et al., 2021). When the sucrose reward was removed, the proportion of flies that showed spontaneous rest behaviour decreased (Wiggin et al., 2021), consistent with reports on increased sucrose intake being associated with increased rest (Murphy et al., 2016) and a sleep suppressing effect of food deprivation (Keene et al., 2010). Therefore, it seems that in this specific task sucrose consumption promotes rest which, in turn, promotes learning.

In terms of the mechanistic process by which sleep facilitates memory acquisition, an attractive hypothesis is that during sleep synapses that have been created during activity in the day are pruned in a process called synaptic homeostasis (Tononi and Cirelli, 2003; Bushey et al., 2011). The idea is that large increases in synaptic strength include the growth of synapses in number and size which is unsustainable because they consume more energy, take up more space, and eventually saturate the coding capacities of the brain, affecting the ability to learn (Bushey et al., 2011). During sleep, synapses are pruned to a sustainable level. This pruning has been reported in a group of cells called the small ventral lateral neurons (LNvs), which are part of the wake promoting system of the circadian network and express the neuropeptide pigment dispersing factor (PDF) (Parisky et al., 2008). Subjecting flies to sleep deprivation and comparing the signal of synaptically-tagged GFP driven under *pdf*-Gal4 in the LNvs between rested and sleep deprived flies showed an increase in presynaptic boutons in the sleep deprived group (Bushey et al., 2011). Similarly, the g neurons of the MB were shown to have larger axonal tips after sleep deprivation, consistent with volume growth of presynaptic terminals, further confirmed by an increase in synaptically-tagged GFP puncta in g neurons in the sleep deprived group (Bushey et al., 2011). An exhaustive analysis of synaptic scaling in memory neurons under sleep deprivation have reported that, although it seems that sleep does generally shape synaptic connectivity in the MB network, the specific effect of the loss of sleep depends on the cell type (Weiss and Donlea, 2021b). Mechanical deprivation of sleep induces an overall increase in the active zone marker Bruchpilot across KCs, which returns to baseline after 48 h of recovery under spontaneous sleep restoration or after 6 h artificially induced sleep through dorsal FB activation (Weiss and Donlea, 2021b). On the other hand, no synaptic changes were detected in PNs or DANs. The analysis of specific KC output synapses onto the sleep inducing MBONg2α’1 using GFP reconstitution across synaptic partners (GRASP) reported a decrease on the connectivity between those neuron types after sleep deprivation (Aso et al., 2014b). This can reflect a decrease on the input weights from the MB to the MBON, but it could also support the idea that, although sleep-inducing, the specific neural pathway involving MBONg2α’1 and the FB is not involved in sleep homeostasis (Dag et al., 2019). It seems, however, that the effects of sleep deprivation in synaptic densities is different depending on the specific cell types involved in those synapses.

Nevertheless, there are lines of evidence supporting the beneficial effects of sleep in synaptic pruning, and eventually favoring memory acquisition. Exposure to complex social interactions leads to synaptic upscaling in LNvs and increased sleep, which is dependent on genes involved in synaptic plasticity (Ganguly-Fitzgerald et al., 2006). However, if flies are subjected to memory training paradigms shortly after social enrichment, they fail to display long-term memory, perhaps showcasing a synaptic saturation incompatible with memory encoding. Supporting the hypothesis of synaptic homeostasis, sleep induction by FB activation immediately after social enrichment restores the memory phenotype (Donlea et al., 2011).

Sleep deprivation during development can lead to long-term changes in synaptic plasticity (Seugnet et al., 2011). In fact, there seems to be a critical period in development where both sleep and LTM emerge at the same time (Poe et al., 2022). During the second instar larvae stage (L2) sleep is not under circadian control (Szuperak et al., 2018). The connections between clock neurons and arousal promoting neurons are formed in the early third instar stage (L3), which brings arousal under clock control to drive circadian sleep (Poe et al., 2022). Interestingly, L2 larvae do not exhibit LTM, but L3 show strong LTM, consistent with the fact that LTM depends on deep sleep, which is not induced until the neural circuits mediating circadian sleep are functional (Poe et al., 2022).

## 3 Memory impairments are rescued by sleep

Considering the discussed effects of sleep facilitating memory formation, one exciting possibility is that induced sleep could in fact rescue sleep defects in pathological conditions. Two classical memory mutants, the phosphodiesterase mutant *dunce* (*dnc*) and the adenylyl cyclase mutant *rutabaga* (*rut*), display generalized impaired learning and memory (Duerr and Quinn, 1982; Folkers, 1982; Livingstone, 1985; Han et al., 1992; Wustmann and Heisenberg, 1997). It is important to highlight that these mutants do not seem to display sleep phenotypes, however, inducing sleep can rescue memory in *rut* and *dnc* mutants (Dissel et al., 2015; Dissel et al., 2020). Three independent strategies were used to induce sleep: activating the dorsal FB, increasing expression of Fatty acid binding protein (dFabp) and administering GABA-A agonist 4,5,6,7-tetrahydroisoxazolo-[5,4-c]pyridine-3-ol (THIP) (Dissel et al., 2015). Each of these strategies increased sleep in wild-type and mutant flies, and, in turn, rescued *rut* and *dnc* learning impairments in APS and courtship conditioning tasks (Dissel et al., 2015). Learning impairments seen in *rut* mutants during the spatial learning heat maze task can also be rescued by THIP-induced sleep (Melnattur et al., 2021). Importantly, THIP-induced sleep in wild-type flies does not enhance STM in the APS further than the baseline performance (Dissel et al., 2015). Both *dnc* and *rut* mutants showed impaired memory during a heat-box place learning task in which flies learn to avoid an area in a box previously associated with high temperatures (Dissel et al., 2020). Induced sleep via THIP rescued performance in *rut* mutants, however, the performance index for *dnc* mutants remained low even after THIP-induced sleep (Dissel et al., 2020). These differences could be due to the particular requirements of cAMP-mediated signalling in a specific task; the circuits that control behavioural performance for different types of memory are different, and they might rely of different genes for different functions (Dissel et al., 2020).

The report that sleep can restore memory defects in mutants that display relative normal sleep phenotypes opened the possibility that sleep could in fact be used for therapeutical purposes in cases of complex disorders that are difficult to treat. This idea has been proposed in clinical settings (Mander et al., 2016). Many neurodegenerative conditions such as Alzheimer’s disease (AD) are associated with both memory defects and sleep disturbances. The *Drosophila* mutant *Presenilin* is used as a model of familial AD. since it recapitulates age-dependent cognitive deficits (Dissel et al., 2015). Young *Presenilin* mutants display normal sleep patterns and intact long-term memory, assessed in courtship conditioning; however, 30-day-old mutant flies have impaired LTM (Dissel et al., 2015). Inducing sleep by administering THIP 2 days prior to and 24 h following training in these aged mutants was sufficient to reverse the LTM deficits in this AD model (Dissel et al., 2015). However, one of the hallmarks of AD in humans is the intercellular accumulation of Aβ plaques (Turner et al., 2003; Zheng and Koo, 2011), therefore several AD models in *Drosophila* aim to replicate this cellular phenotype. Expression of the human Aβ42 peptide in flies leads to the development of key features of AD, like age-dependent learning impairment (Iijima et al., 2004). Interestingly, this model of AD in *Drosophila* showcased a reciprocal relationship between sleep and Aβ pathology: Aβ expression leads to fragmented and reduced sleep, associated with increased neural excitability, and conversely, sleep deprivation enhances the detrimental effects of Aβ accumulation (Tabuchi et al., 2015; Dissel et al., 2017). Enhancing sleep in this case reduced Aβ deposition (Tabuchi et al., 2015). Ubiquitously co-expressing the human amyloid precursor protein and β-secretase (APP:BACE) in adult flies also results in the accumulation of Aβ peptides and memory impairment (Chakraborty et al., 2011). Induced sleep in APP:BACE 14-day old flies rescued short-term memory impairments in the APS task and long-term courtship conditioning defects. Moreover, 2-days of induced sleep reduced C-terminal Aβ peptide accumulation, and reversed synaptic deficits of APP:BACE flies (Dissel et al., 2017).

It seems then that a better understanding of this relationship between sleep and pathology can advance neurological recovery. It has been previously reported that sleep can benefit memories by decreasing forgetting. In humans it has been proposed that sleep benefits both declarative and non-declarative memories in two very different ways: non-declarative memories, which are independent of hippocampal function, are often enhanced after a period of rapid eye movement (REM) sleep. In this case, the memory performance increases over the initial level. By contrast, hippocampus-dependent declarative memories benefit from sleep by reducing retroactive interference. In other words, there is decreased forgetting during non-REM sleep. This hypothesis has been called the “opportunistic theory of cellular and systems consolidation”. There is evidence of this opportunistic theory in *Drosophila*, Berry et al. (Berry et al., 2015) recently discovered that a small group of DAN functionally connected to KC mediates the process of active forgetting of olfactory memories. Blocking synaptic output from these cells increases memory retention. Conversely, stimulating their output after learning increases memory loss. Interestingly, these DAN are the same neurons that convey the punishment signal during the acquisition of aversive memory (Schwaerzel et al., 2003; Schroll et al., 2006; Claridge-Chang et al., 2009; Aso et al., 2010; Aso et al., 2012; Berry et al., 2012). Moreover, functional imaging of these DAN demonstrated the presence of chronic calcium activity before and after training. This chronic dopaminergic activity is modulated by the animal's behavioral state (Berry et al., 2015). Strong activity is observed during periods of high locomotion, and little activity is observed during rest or sleep periods. Furthermore, increasing pharmacological or genetic sleep induction decreases chronic dopaminergic activity while enhancing memory retention. Contrarywise, artificial or mechanical increase of arousal stimulates chronic dopaminergic activity and accelerates DA-based forgetting (Berry et al., 2015). Recently, a model of AD in *Drosophila* mimicked early-stages of the disease by restricting the expression of the Arctic variant of the Aβ42 peptide (Aβ^Arctic^) to the MB. These genetic modification resulted in normal learning in both aversive and appetitive olfactory associative conditioning tasks, but lead to what could be considered accelerated forgetting (Kaldun et al., 2021). In both STM and LTM olfactory associative conditioning tasks flies expressing Aβ^Arctic^ in MB displayed similar learning scores immediately after training than their controls, but there was a significant memory loss after 2 h in STM tasks, and after 6 h in LTM tasks. Although it is difficult to discard an effect of deficient memory consolidation, this memory phenotype can be reversed by silencing the aforementioned specific DANs involved in the active forgetting process (Berry et al., 2015). Inducing sleep by both activating the FB after training and pharmacologically via THIP ingestion can restore the rate of forgetting in flies expressing Aβ^Arctic^ in the MB (Kaldun et al., 2021). Hence, MB neurons expressing Aβ^Arctic^ could influence the network increasing the activity of forgetting DANs, process that can be suppressed by induced sleep.

## 4 Sleep in memory consolidation

Memory consolidation is the process of converting new initially labile memories into robust, long-lasting protein synthesis-dependent memories (Roselli et al., 2021). The consolidated memories are then integrated into a complex memory structure composed of all previous relevant memories. It has been suggested that the consolidation of memories requires the reactivation of neuronal activity patterns within the neural circuits initially active during the acquisition of new memories. The best example of this is the replay of place cells. Lee and Wilson (Lee and Wilson, 2002) showed that firing sequences of place cells during the walking of simple trajectories were replayed in the hippocampus offline. It is plausible that this replay selectively strengthens memory-specific cell assemblies that promote memory consolidation.

Furthermore, the electrical disruption of these offline replay events reduces the learning rate of a spatial memory task (Girardeau et al., 2009; Ego-Stengel and Wilson, 2010). Remarkably, the consolidation process occurs mainly during sleep. The presence of time-compressed versions of place cell sequences that correspond to preceding trajectories of the animals, particularly during sharp-wave ripples, has been reported (Lee and Wilson, 2002; Diba and Buzsaki, 2007; Davidson et al., 2009). It is plausible that the memory consolidation process is favored during the offline time because encoding and retrieval of memories occurs during wake, and they, presumably, could cause catastrophic interference and impede memory consolidation.

In *Drosophila*, the role of post-learning sleep on memory consolidation has also been documented. Memory consolidation has been better characterized in olfactory memories. During classical olfactory conditioning, the synaptic output of the dorsal paired medial neurons (DPM) is required for memory consolidation (Waddell et al., 2000; Yu et al., 2005; Lee et al., 2011; Pitman et al., 2011; Cervantes-Sandoval and Davis, 2012; Lee et al., 2021; Muria et al., 2021). DPMs are a pair of serotonergic neurons innervating the MB (Lee et al., 2011; Pitman et al., 2011; Cervantes-Sandoval and Davis, 2012). It was first reported that blocking the synaptic output of DPM neurons after learning but before memory retrieval impaired memory consolidation (Keene et al., 2006). Remarkably, DPM neurons showed learning-induced plasticity, characterized by increased calcium responses to odors paired with either electric shock or sugar reward (Yu et al., 2005; Pitman et al., 2011; Cervantes-Sandoval and Davis, 2012). The duration of these changes correlated with the strength of the memory. STM will induce a transient increase in odor responses, whereas LTM will induce lasting changes (Cervantes-Sandoval and Davis, 2012). Optical analysis of DPM anatomy and, more recently, the connectome data, agree with the idea that DPM forms a recurrent feedback loop with KCs in the MB, continual activation of which favors consolidation of memory (Pitman et al., 2011; Takemura et al., 2017; Li et al., 2020). Despite the clear role of DPM neurons and memory consolidation, a link between DPM function and the “need for sleep” for consolidation remained elusive. Later, however, evidence indicated that DPM activity serves as a link between sleep and memory consolidation. Haynes et al., (Haynes et al., 2015), reported that in addition to being serotonergic, DPMs are also inhibitory GABAergic neurons. The authors demonstrated that artificial thermogenetic activation of DPM neurons robustly promotes sleep. Furthermore, they showed that inhibiting both GABA and serotonin synthesis in DPM neurons resulted in decreased nighttime sleep. They demonstrated that DPMs are sleep-promoting by inhibiting the activity of wake-promoting α’/β’ KC neurons. Using functional imaging with SuperClomeleon reporter, it was demonstrated that artificial chemoactivation of DPM neurons induced chloride influx in α’/β’ KC neurons. Furthermore, knocking down the GABA receptor *Rdl* in α’/β’ KC neurons resulted in decreased sleep.

**FIGURE 1:**
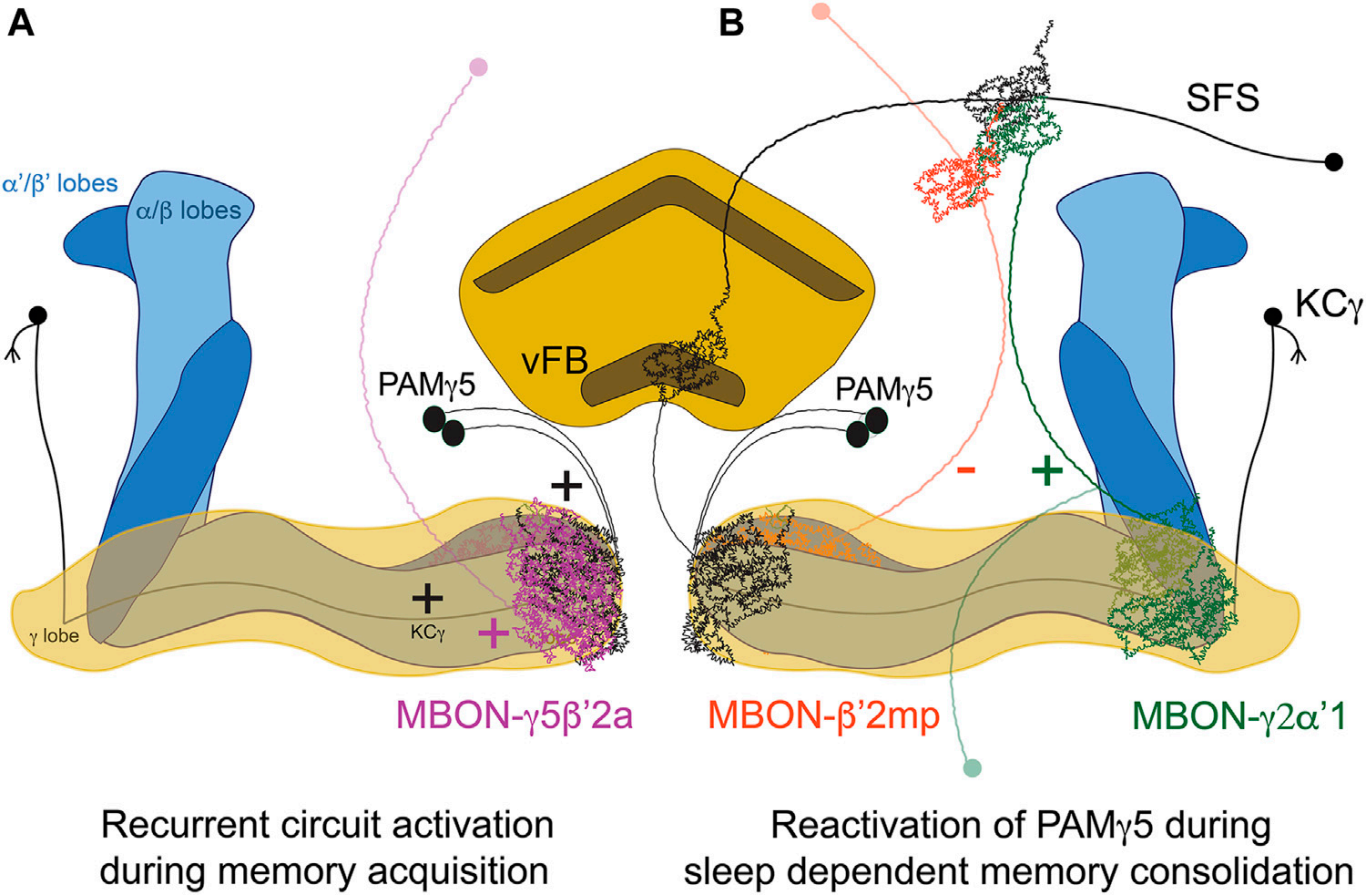
Circuit for sleep-dependent consolidation of courtship memory. **(A)** Recurrent activation of the microcircuit formed by KCγ, MBON-γ5β’2a and PAMγ5 leads to prolonged activation of the DANs, levering memory formation. **(B)**. MBON-γ2α’1 activates the vFB through SFS neurons, promoting sleep and memory consolidation. vFB projections to KC-γ5 mediate circuit reactivation during sleep. In contrast, the activity of MBON-β’2mpduring STM-inducing protocols inhibits SFS/vFB and promotes wakefulness.

The neural substrates mediating the effect of sleep in memory consolidation have also been extensively studied in the case of courtship memory in flies. Naïve male flies will actively court both virgins and mated females, while males exposed to and rejected by unreceptive mated females will subsequently become less prone to court receptive virgin females (Siegel and Hall, 1979). This reduction in the courtship index reflects the newly acquired memory, which is dependent on the MB (McBride et al., 1999). Courtship training for shorter times leads to STM that can last hours, whereas longer training sessions will induce protein-synthesis dependent long-term memory. MB KC-γ neurons are essential for the formation of courtship memories (Keleman et al., 2012). Similarly, acute inhibition of MB output neuron MBON-γ5β’2α (also known as M6), which is postsynaptic to KC-γ, both during acquisition and during memory retrieval, impairs memory (Zhao et al., 2018). This potentiation is modulated by dopaminergic input from PAM-γ5 (also known as aSP13). Moreover, MBON-γ5β’2α synapses back into PAM-γ5 (Figure 1). This suggests the presence of a recurrent feedback loop that reactivates the neural learning pathways during memory consolidation. But how does sleep modulate this circuit? Fascinatingly, sleep drive is plastic and is modulated by experience. Males trained with paradigms that form LTM sleep more after learning than untrained controls, or counterparts subjected to STM-inducing protocols (Ganguly-Fitzgerald et al., 2006; Dag et al., 2019), pointing at a demand of sleep after complex learning experiences. This “need for sleep” will enable and engage consolidation mechanisms, as proven by studies showing that sleep deprivation after training abolishes LTM (Ganguly-Fitzgerald et al., 2006; Donlea et al., 2011; Dag et al., 2019). A recently published study identified specific neural circuits mediating this induction of sleep exclusively after LTM-inducing training protocols, and not after shorter training ones. The timing of the serial activation of two MBONs switches on/off sleep driving circuits in the FB (Lei et al., 2022). MBON-γ2α’1 activates sleep-inducing ventral FB (vFB) neurons. The activity of MBON-γ2α’1 increases proportionally with the length of the training session, and synaptic release from this neuron is necessary for both the increase of sleep post-training and the consolidation of memory (Lei et al., 2022). On the other hand, MBON-β’2mp suppresses sleep (Aso et al., 2014b), inhibits vFB neurons, and displays higher activity levels with shorter, STM-inducing training protocols (Lei et al., 2022). These two MBON form a polysynaptic circuit with vFB; they feed directly into SFS neurons which integrate both MBON signals and then convey excitatory input to vFB. These findings lever an elegant model where STM-inducing protocols will recruit MBON-β’2mp, which inhibits the vFB facilitating wakefulness and, on the other hand, LTM-inducing protocols recruit MBON-γ2α’1, which activates the vFB promoting sleep and memory consolidation.

Important studies in the field have identified distinct FB-neural populations that seem to induce post-training sleep with different rules, time scales and circuits. Dorsal FB-neurons (dFB) are involved in sleep homeostasis (Donlea et al., 2011; Donlea et al., 2014) and have been reported in a seminal study to promote sleep and memory consolidation when activated for 4 h immediately after training, even when the males were trained in short-lasting sessions that would not induce LTM normally (Donlea et al., 2011). Importantly, this protocol of dFB activation was not enough to consolidate memory when combined with sleep deprivation, which points to a specific role of dFB driving sleep, and not general activation of the circuit, as the main factor for memory consolidation. More recently, the vFB has been proposed as a key brain area responsible for learning-induced sleep and memory consolidation (Dag et al., 2019). The vFB also induces sleep, and its activation during a specific time window of 5–7 h after the onset of training is sufficient to consolidate memory when the flies where subjected to short training sessions that would not induce LTM normally (Dag et al., 2019). vFB neurons are active after LTM-training during consolidation, and they project to the aforementioned PAMγ5. Interestingly, these dopaminergic neurons have been reported to promote wakefulness and reduce sleep (Sitaraman et al., 2015b; Driscoll et al., 2021), which could reflect the importance of previous experience (mediating pre-existing plasticity) or the activation kinetics of neural activity in subsequent behavioral outputs. Nevertheless, sleep mediated by the vFB is the factor that mediates circuit reactivation during sleep involving the feedback loop between PAMγ5, MBγ neurons and MBON-γ5β’2α (Zhao et al., 2018), which reflects sleep-dependent memory consolidation models in mammals (Diekelmann and Born, 2010). The availability of genetic access to discrete neural subpopulations of the FB and their post-synaptic partners will further enable the dissection of the circuits mediating sleep and courtship memory consolidation with unprecedented resolution.

Altogether this evidence shows that memorable experiences increase the sleep drive. During sleep, there is a reactivation of the neuronal pathways activated during learning. This favors the transition from short-term labile and transient memories into persistent LTM.

## 5 Olfactory appetitive conditioning and sleep-independent consolidation

**FIGURE 2:**
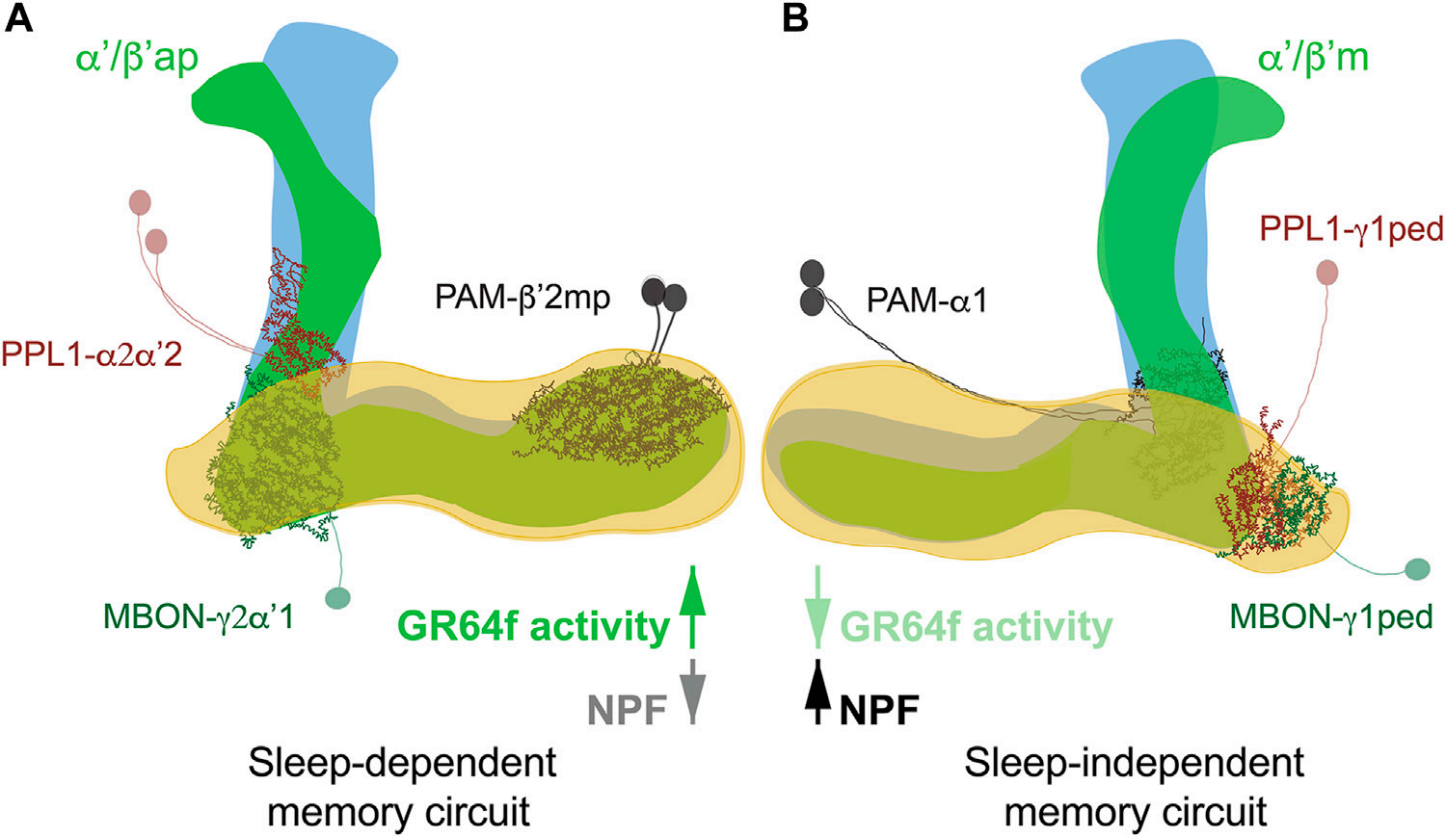
Sleep-dependent **(A)** and sleep-independent **(B)** circuits for appetitive memory. Fed flies form sleep-dependent memory that requires activity in α′/β′ap neurons in association with a circuit comprised of PAM-β′2mp and recurrent connections between PPL1-γ2α′1 DANs and MBON-γ2α″1. In contrast, sleep-independent long-term memory in starved flies is mediated by α′/β′m neurons interacting with PAM-α1 and reciprocal signalling between PPL1-γ1pedc DANs and MBON-γ1pedc. The switch between circuits is mediated by sweet taste signals received by gustatory receptor GR64f in fed flies and hunger signals mediated by NPF in starved flies.

The metabolic state, i.e. satiation or starvation, is paramount for memory formation. Expression of food-associated memory is promoted by hunger and inhibited by satiety (Krashes et al., 2009). Typically, in appetitive conditioning assessments, flies are starved after training to enable memory retrieval (Krashes and Waddell, 2008). It was found, though, that depending on the post-training metabolic state appetitive memory has different requirements for sleep and different neuronal circuits are involved (Figure 2) (Chouhan et al., 2021; Chouhan and Sehgal, 2022). Post-training feeding promotes a sleep-dependent type of learning; while starvation triggers a consolidation mechanism which is sleep-independent (Chouhan et al., 2021). Unexpectedly, it is not the caloric intake of sugar but its sweetness that promotes the switch between sleep-dependent or independent memory. In fact, trained flies kept on arabinose (a sweet but non-nutritious sugar) form sleep-dependent memories (Chouhan et al., 2021), while trained flies kept on sorbitol (a tasteless but nutritious sugar) do not require sleep for memory formation (Chouhan and Sehgal, 2022). Sweet taste signals the presence of food, determines the initial feeding preferences of the animal (Stafford et al., 2012), and may signal reward through the activation of PAM DANs (Chouhan and Sehgal, 2022). It also seems to be responsible for the memories to switch between sleep-dependent and independent consolidation. In fact, enhancing activity of sweet-sensing neurons (Gr64f+) in starved flies induces a switch from sleep-independent to a dependent form of memory (Chouhan and Sehgal, 2022). The opposite is true for fed flies, in which inactivation of sweet-sensing neurons causes the switch from sleep-dependent to independent memory formation.

Molecularly the switch is mediated by neuropeptide F (NPF) (Chouhan et al., 2021). NPF is a hunger signal (Wu et al., 2003; Wu et al., 2005), and its expression in neurons is indicative of food deprivation (Krashes et al., 2009). When starved flies, which usually express NPF, have a lack of NPF or its receptor, sleep-dependent memory consolidation is activated (Chouhan et al., 2021), supporting the idea that NPF mediates the switch between sleep-independent to sleep-dependent memories flies.

At circuit level α’/β’ lobes of the MB are known to be required for appetitive memory (Krashes and Waddell, 2008) and to drive wakefulness (Haynes et al., 2015; Sitaraman et al., 2015b). In fed flies, sleep-dependent consolidation of memory is mediated by α’/β’ anterior-posterior (α’/β’ap) neurons, which increase sleep when active. While in starved flies sleep-independent memory consolidation is mediated by α’/β’medial (α’/β’m) neurons, which reduces sleep when active (Chouhan et al., 2021). Interestingly, inactivation of both neuronal subtypes, before training, doesn’t affect sleep, maybe because both those neuronal subtypes induce sleep in the specific context of appetitive conditioning. As expected, sleep loss in fed flies reduces Ca^2+^ signal in α’/β’ap neurons, while blocking α’/β’ap neuronal transmission post-training decreases sleep amount and bout length (Chouhan et al., 2021), arguing that α’/β’ap are required for post-training sleep increase in fed flies, while α’/β’m neurons are dispensable.

Consolidation of appetitive memories requires, at least, two other neuronal populations: DANs and MBONs (Aso et al., 2014b; Musso et al., 2015; Aso and Rubin, 2016). Sleep-dependent and independent consolidation activates and requires distinct neuronal circuits for memory formation. Sleep-independent consolidation occurs in animals that are starved post-training and requires α’/β’ m, PPL1-γ1ped (also known as MB-MP1) (Musso et al., 2015), PAM-α1 (Ichinose et al., 2015) and MBON-γ1pedc (Pavlowsky et al., 2018). Sleep-dependent consolidation of memory, on the other hand, requires the synergistic action of α’/β’ap neurons and the recurrent circuit PPL1-γ2α’1 MBON-γ2α’1 PPL1-γ2α’1. PPL1-γ2α’1 (also known as MB-MV1), projects to the sleep-promoting neurons MBON-γ2α’1 (Felsenberg et al., 2017; Berry et al., 2018), which subsequently recruits PAM-β’2mp, essential for LTM in fed flies (Chouhan and Sehgal, 2022). Interestingly, PAM-β’2mp project to the aforementioned MBON-γ1pedc which are essential for memory in starved animals (Aso et al., 2014b; Li et al., 2020).

As a mechanism, it is proposed that sweet taste activates PAM-β’2mp as reward signal. PAM-β’2mp then engage with MBON-γ2α’1 to establish sleep-dependent memory. On the other hand, PAM-β’2mp response might also be modulated by MBON-γ1pedc. MBON-γ1pedc might inhibit PAM-β’2mp to allow PAM-α1 to form sleep-independent memories (Chouhan and Sehgal, 2022).

## 6 Conclusion

Memory can be affected by numerous biological processes, and sleep might be one of the most relevant ones, due to its universality and drastic effects. As reviewed here, memory is impaired by sleep deprivation, and memory defects can be rescued by inducing sleep in models of pathological neurodegeneration, such as AD. These findings support potential therapeutic benefits to mechanistically understanding the interaction between memory and sleep. It will be interesting to assess how general this beneficial function of sleep is in neurological processes, and if and how it could enhance cognitive brain function in cases of acquired damage, for example.

Recent studies have tremendously advanced our understanding of the circuit motifs that mediate sleep dependent memory consolidation. In-depth analysis of the neural subpopulations of the FB with new and better drivers will continue to decipher the specific contributions of the vFB and peripheral neurons with emerging described roles in sleep to cognitive processes with unprecedented resolution. Similarly, circuit dissection strategies can unravel the mechanisms of sleep independent memory consolidation and how general they are. It will also be interesting to establish the role glia has in the interaction between sleep and memory, particularly in models of AD.

However, there is evidence of large variability on the effect of sleep in memory in *Drosophila* (Weiss and Donlea, 2021a), depending on the metabolic state, genetic background or the specific type of memory. With the appropriate technical development, it is critical to perform sleep deprivation experiments using devices that keep the animals awake while imparting the least stress and mechanical damage as possible. By doing so, we now know that there is a wide variety of sleep duration between individuals, with some flies showing as little as 4 min of spontaneous sleep per day (Geissmann et al., 2019). In this study, it was found that even when subjecting flies to life-long sleep deprivation there is no evidence of a difference in lifespan between these sleep deprived flies and the control group (Geissmann et al., 2019). It will be exciting to investigate spontaneous short sleeping flies and sleep deprived ones to establish whether they suffer from cognitive defects such as memory impairments, and to assess if they can undergo canonical processes of memory consolidation.

## Glossary

AD: Alzheimer’s Disease
APP: β-Amyloid Precursor Protein
Appl: β-Amyloid Precursor Protein-like
APS: Aversive Phototaxic Suppression
BACE: β-Site Amyloid precursor protein Cleaving Enzyme
DA1: D1-dopamine receptor
DAN: Dopaminergic Neuron
DART: Drosophila ARousal Tracking system
dEaat1: *drosophila* Excitatory amino acid transporter 1
dFabp: *drosophila* Fatty Acid Binding Protein
DPM: Dorsal Paired Medial
EB: Ellipsoid Body
FB: Fan-shaped Body
GRASP: GFP Reconstitution Across Synaptic Partners
L2: Second instar larva
L3: Third instar larva
LNvs: Small Ventral Lateral Neurons
LTM: Long-Term Memory
MBON: Mushroom Body Output Neuron
NPF: Neuropeptide F
PAM: Protocerebral Anterior Medial
PDF: Pigment Dispersing Factor
PER: Proboscis Extension Reflex
PPL1: Protocerebral Posterior Lateral 1
PPL2: Protocerebral Posterior Lateral 2
REM: Rapid Eye Movement SEZ: Subesophageal Zone
SLIDE: Sleep Interrupting Device
SNAP: Sleep Nullifying Apparatus
STM: Short-term Memory
THIP: GABA-A agonist 4,5,6,7-tetrahydroisoxazolo-[5,4-c]pyridine-3-ol (Gaboxadol).
